# Critical Role of Molecular-Based Stratification in Low-Risk Myelodysplastic Syndrome with Direct Progression to Acute Myeloid Leukemia: A Case Report

**DOI:** 10.3390/ijms27104557

**Published:** 2026-05-19

**Authors:** Stejara Nicoleta Mihai, Denisa Dragu, Cristina Mambet, Anca Botezatu, Petruta Gurban, Laura G. Necula, Lilia Matei, Ana Iulia Neagu, Ioana Pitica, Marius Ataman, Saviana Nedeianu, Mihaela Chivu-Economescu, Coralia Bleotu, Catalina Roxana Grosu-Ferea, Cristina Ciufu, Carmen C. Diaconu, Ana Maria Vladareanu

**Affiliations:** 1 Faculty of Medicine, University of Medicine and Pharmacy Carol Davila Bucharest, 050474 Bucharest, Romania; stejaramihai@yahoo.com (S.N.M.); cristina.mambet@virology.ro (C.M.); ana.neagu@virology.ro (A.I.N.); cciufu@yahoo.com (C.C.); anamaria.vladareanu@umfcd.ro (A.M.V.); 2 Laboratory of Medical Analyses, Hematology, Dr. Carol Davila Central Military Emergency University Hospital, 010825 Bucharest, Romania; roxana.ferea@gmail.com; 3 Department of Cellular and Molecular Pathology, Stefan S. Nicolau Institute of Virology, Romanian Academy, 030304 Bucharest, Romania; laura.necula@virology.ro (L.G.N.); lilia.matei@virology.ro (L.M.); ioana.pitica@virology.ro (I.P.); marius.ataman@virology.ro (M.A.); saviana.nedeianu@virology.ro (S.N.); mihaela.economescu@virology.ro (M.C.-E.); coralia.bleotu@virology.ro (C.B.); carmen.diaconu@virology.ro (C.C.D.); 4 Hematology Department, University Emergency Hospital of Bucharest, 050098 Bucharest, Romania; 5 Molecular Virology Department, Stefan S. Nicolau Institute of Virology, Romanian Academy, 030304 Bucharest, Romania; anca.botezatu@virology.ro; 6 Cytogenomic Medical Laboratory, 014453 Bucharest, Romania; petruta_gurban@yahoo.com; 7 Hematology Bone Marrow Transplant, University Emergency Hospital of Bucharest, 050098 Bucharest, Romania

**Keywords:** myelodysplastic syndrome, acute myeloid leukemia, next-generation sequencing, single nucleotide polymorphism array, risk stratification, homozygous *TET2* variants, case report

## Abstract

The genomic landscape of myelodysplastic syndromes/neoplasms (MDS), a heterogeneous group of myeloid malignancies defined by bone marrow cell dysplasia with ineffective hematopoiesis, includes somatic and, less frequently, germline mutations in hematopoietic stem and progenitor cells, along with chromosomal abnormalities. The latest World Health Organization 2022 classification of myeloid neoplasms, as well as stratification in lower-risk (LR) and higher-risk (HR) MDS using either the Revised International Prognostic Scoring System (IPSS-R) or the Molecular International Prognostic Scoring System (IPSS-M), guide prognostic assessment and risk-adjusted therapy. We report the case of an 81-year-old patient diagnosed with LR-MDS according to IPSS-R that exhibited direct progression to acute myeloid leukemia. The retrospective analysis of paired DNA samples from MDS and leukemic phases, obtained four months apart, using both targeted next-generation sequencing and single nucleotide polymorphism array, indicated swift alterations in the genomic profile, being suggested that the leukemic clone emerged from the clone harboring homozygous *TET2* and heterozygous *SRSF2* variants that acquired *RUNX1*, *BCOR*, *BCORL1* likely pathogenic mutations and trisomy 13. By employing IPSS-M for prognostic evaluation at the MDS phase, the patient would have been assigned to the HR-MDS category with a possible benefit from hypomethylating agent therapy. Risk stratification is of pivotal importance in a patient-centered approach to MDS treatment being significantly improved by incorporating the molecular genetic findings.

## 1. Introduction

Myelodysplastic syndromes/neoplasms (MDS) include a heterogeneous group of malignancies defined by bone marrow (BM) cell dysplasia with ineffective hematopoiesis resulting in various peripheral blood (PB) cytopenias and carrying an increased risk of transformation into acute myeloid leukemia (AML) [1].

MDS incidence increases with age, being highly suspected in patients with unexplained anemia or, less frequently, isolated thrombocytopenia or neutropenia, bicytopenia, or pancytopenia. Fatigue is a common symptom, while recurrent infections or mucocutaneous bleeding may be experienced occasionally [1,2].

For establishing MDS diagnosis, it is mandatory to rule out non-neoplastic causes of cytopenias and perform BM morphologic examination, as well as conventional cytogenetics [2].

In many cases, MDS is preceded by an age-related asymptomatic state, namely clonal hematopoiesis of indeterminate potential (CHIP). Somatic mutations involving epigenetic regulatory genes, such as *DNMT3A*, *TET2*, and *ASXL1*, are typically found in this condition. A causal relationship between aging, CHIP mutations, inflammation, changes in the BM microenvironment, and MDS development has been recently defined [3].

The genomic landscape of MDS comprises somatic and, less frequently, germline mutations in hematopoietic stem and progenitor cells (HSPCs), along with chromosomal abnormalities, displaying prognostic and therapeutic relevance [4,5]. This is reflected in the latest World Health Organization (WHO) 2022 classification of myeloid neoplasms that recognized as separate entities in the larger category of MDS with defining genetic abnormalities the following subtypes: MDS with isolated 5q deletion that benefits from lenalidomide therapy, MDS with *SF3B1* mutation (or ≥15% BM ring sideroblasts—RS) that generally has a favorable outcome, and MDS with *TP53* biallelic inactivation that associates a poor prognosis [6,7].

Several prognostic classifications have been developed for MDS patients; the most commonly applied being the Revised International Prognostic Scoring System (IPSS-R) that considers the percentage of BM blasts, the PB cytopenias, and the cytogenetic risk group. The recently proposed molecular IPSS (IPSS-M) has improved risk assessment by incorporating molecular aberrations detected by next-generation sequencing (NGS) assays [8]. For risk-adapted therapy, the stratification in lower-risk (LR) and higher-risk (HR) MDS using either IPSS-R or IPSS-M is critical. While in LR-MDS the therapy focuses mainly on anemia alleviation to reduce the transfusion needs, in HR-MDS the therapeutic approaches aim to prolong survival by using hypomethylating agents (HMAs) [7].

During the natural course of disease approximately 30–40% of MDS patients will experience leukemic transformation. However, the complications related to cytopenias often occurring on the background of various comorbidities represent an important cause of non-AML mortality, especially in LR-MDS patients. A recent study has delineated patterns of disease progression in LR-MDS, highlighting the clinical and molecular characteristics of those patterns [9]. From the clinical and routine laboratory perspective, male gender, neutropenia, thrombocytopenia, increased percentage of BM blasts, ferritin > 1000 µg/L, albumin < 3.5 g/dL, multilineage dysplasia, and lack of RS were identified as risk factors of progression to HR-MDS or AML [9].

In this report, we describe the case of an elderly male patient diagnosed with LR-MDS with RS that progressed directly to AML. A retrospective analysis of paired DNA samples from the MDS and leukemic phases, by both targeted NGS and single nucleotide polymorphism (SNP) array, was performed, aiming to highlight the mechanisms of AML development from LR-MDS.

## 2. Case Presentation

An 81-year-old man known with the β-thalassemia trait received the diagnosis of LR-MDS with multilineage dysplasia (IPSS-R = 3) in March 2018, displaying severe anemia (Hb 7.3 g/dL), moderate thrombocytopenia (62 × 10^9^/L), normal white blood cell count (WBC) with normal absolute neutrophil count (ANC), hypercellular bone marrow with dyserythropoiesis, dysmegakaryopoiesis and 2% blasts, along with a normal karyotype. The recommended treatment consisted of weekly administration of epoetin alfa, the Hb level being maintained between 8.3 and 8.9 g/dL. In August 2021, the patient was admitted at the Hematology Clinic of the University Emergency Hospital Bucharest for marked asthenia related to severe anemia (Hb 5.1/dL). CBC also indicated moderate neutropenia (ANC 0.9 × 10^9^/L) and thrombocytopenia (PLT 50 × 10^9^/L). A BM aspirate was obtained indicating a hypercellular BM, with erythroid hyperplasia, dyserythropoiesis, dysmegakaryopoiesis and 1–2% blasts. Perls stain revealed 45% ring sideroblasts (RSs). A normal karyotype was detected at routine cytogenetics. Fluorescence in situ hybridization panels for MDS were not available. At this moment, the patient still belonged to the LR-MDS category (IPSS-R = 3); however, he became transfusion dependent, receiving monthly red blood cell (RBC) transfusions in addition to epoetin alfa therapy. Four months later, the patient presented at the Hematology Clinic for the periodic RBC transfusion complaining of dyspnea and nausea. Automated CBC and PB examination were consistent with the leukemic transformation of MDS: WBC 102.6 × 10^9^/L with 91% blasts, Hb 7.2 g/dL, PLT 36 × 10^9^/L. The immunophenotyping of blasts by flow cytometry established the diagnosis of AML without maturation. Due to rapid alteration of the patient’s general condition with severe neurologic and respiratory manifestations of leukostasis, bone marrow aspiration could not be performed. Therefore, appropriate samples for conventional cytogenetics were not available. Hydroxyurea was given to lower the WBC count in conjunction with supportive therapy. Disease evolution was fulminant, with progressive respiratory failure and death within two days from admission. A summary of the laboratory findings during the MDS and AML phases is shown in Table 1.

We analyzed retrospectively the available DNA samples from August 2021 and December 2021, respectively, to emphasize the genetic factors involved in MDS progression directly into AML during a short interval. The research was conducted at Stefan S. Nicolau Institute of Virology in compliance with the Declaration of Helsinki and approved by the local ethics committee (No. 136/06.02.2017 rev. no.131/18.01.2019). A separate written informed consent was obtained from the patient at the moment of blood and bone marrow collection for the genetic analyses. For all subsequent analyses, patient-specific information was de-identified.

Peripheral blood mononuclear cells (PBMCs) and bone marrow mononuclear cells (BMMCs) were obtained by density gradient centrifugation using Ficoll-Paque PREMIUM sterile solution (GE Healthcare Life Sciences, Little Chalfont, UK) according to standard procedures. DNA was extracted from PBMCs and BMMCs using the PureLink™ Genomic DNA Mini Kit (ThermoFisher Scientific, Waltham, MA, USA).

In the first stage, we analyzed the paired DNA samples obtained from BMMCs (MDS phase) and PBMCs (consisting almost exclusively of blasts in the leukemic phase) by targeted NGS, employing the TruSight Myeloid Sequencing Panel (Illumina, San Diego, CA, USA), an amplicon-based NGS panel that covers the full exonic regions of 15 genes and exonic hot spots of 39 genes strongly associated with myeloid malignancies. Interpretation of sequence variants was performed in conformity with the Association for Molecular Pathology (AMP) and the American College of Medical Genetics and Genomics (ACMG) guidelines [10]. At the MDS phase, a frameshift *TET2* variant—*TET2* c.4317dup, p.R1440Tfs*38 (COSV54401441), tier 2 variant according to AMP, likely pathogenic according to ACMG classification, with variant allele frequency (VAF) of 53.6% along with an in-frame deletion in *SRSF2* gene c.284_307del, P95_R102del, tier 3 variant according to AMP and variant of unknown significance (VUS) according to ACMG, with a VAF 25% were detected. At the leukemic phase, a significant increase in VAF of *TET2* and *SRSF2* variants was noticed, 94.1% and 50.2% respectively, and three novel unreported variants were identified: frameshift *RUNX1* c.504_505del p.G170Efs*42 with VAF of 39%, and nonsense variants *BCOR* c.626C>A, p.Ser209* and *BCORL1* c.4012C>T, p.Arg1338* with VAF of 85.9% and 86.2%, respectively. According to AMP/ACMG classification, the *RUNX1* variant is considered tier 1 and “likely pathogenic”, while *BCOR* and *BCORL1* variants are classified as tier 3 “likely pathogenic”; targeted NGS results are cumulated in Table 2.

**Figure 1:**
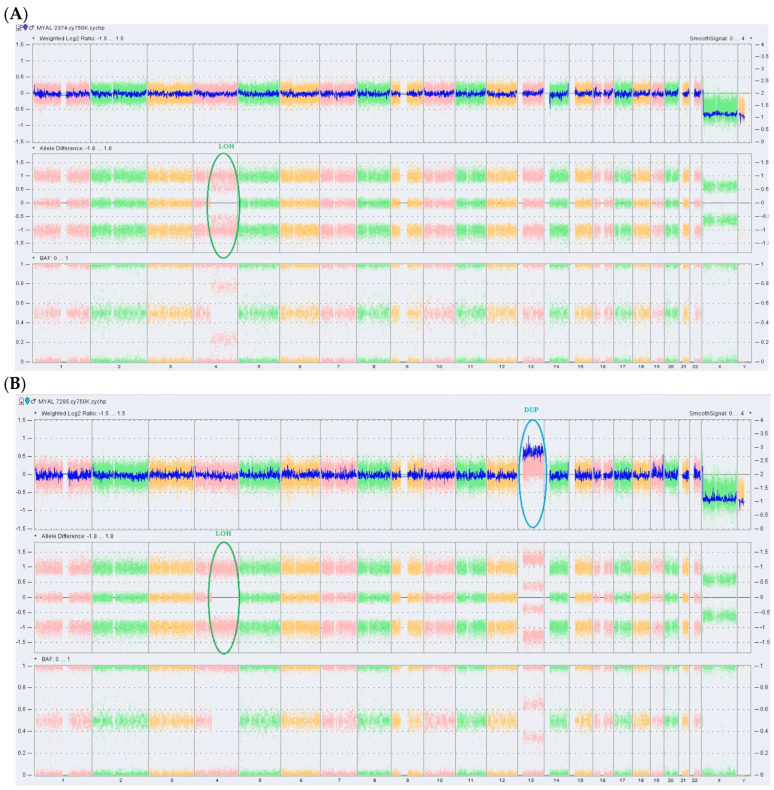
Single nucleotide polymorphism microarray results in MDS patient during MDS phase showing no gain or loss of genetic material (**A**), and in the leukemic phase showing a gain of genetic material (**B**).

In the second stage, the same DNA samples were analyzed for genome-wide presence of copy number variations (CNVs) and copy-neutral loss of heterozygosity (CN-LOH) using the CytoScan 750K Array on the GeneChip^®^ System 3000 instrumentation platform (Affymetrix, Santa Clara, CA, USA) that ensures a full exon coverage of 526 cancer-related genes. Chromosome Analysis Suite Software v4.2.1 (ThermoFisher Scientific) was used for data analysis. As shown in Figure 1A, no CNVs were detected at the MDS phase; instead, a region of CN-LOH, at chromosome 4q13.3q35.2, covering 116.81 Mb was observed. This anomaly is a mosaic CN-LOH affecting approximately 70% of the analyzed BMMCs and comprising 374 genes, including *TET2* found on chromosome 4q24. At the AML phase, the CN-LOH anomaly was present in all analyzed cells and, additionally, a gain of 95.67 Mb at region 13q11q34 (suggestive for trisomy 13) was identified.

Analysis of CNVs and CN-LOH at the level of whole genome using Chromosome Analysis Suite Software v4.2.1 in DNA samples obtained at the MDS phase (A) and at transformation into secondary AML (B) was conducted. Each SNP in the array is represented by a unique dot in the plots. In sample A, the highlighted CN-LOH at chromosome 4q is partial, being present in ~70% of cells (mosaic LOH), while in sample B this event affects all analyzed mononuclear cells. Allele difference and B allele (BAL) plots indicate an allelic imbalance (two tracks instead of the normal three tracks) consistent with the LOH aberration. In Figure 1A, the weighted log2 is centered around the 0 line (no gain/loss of genetic material); in Figure 1B, the weighted log2 is shifted above the 0 line on chromosome 13q corresponding to a gain of genetic material at this level.

**Figure 2:**
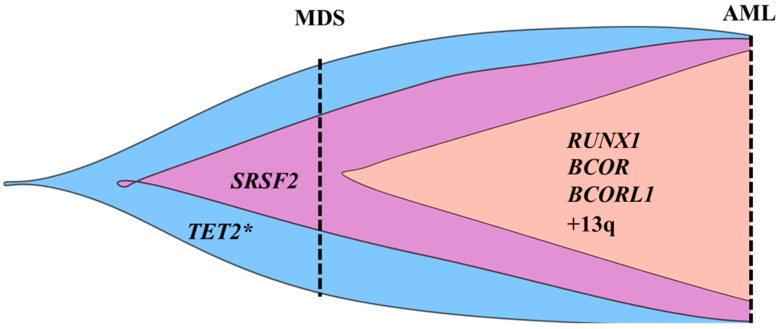
Graphical representation of the hypothetical clonal evolution pattern in MDS patient. A linear pattern of clonal evolution inferred from VAF of the somatic mutations detected by targeted NGS and from data provided by SNP array is observed. Vertical dashed lines mark the sampling moments. As DNA genotyping from single colonies could not be performed, the order of mutation acquisition and subclonal architecture were presumed from the mutational loads. * Indicates a mutated *TET2* gene that is also affected by CN-LOH. Created in BioRender. MAMBET, C. (2026) https://BioRender.com/oqmxn7f (accessed on 14 May 2026).

The analysis of paired DNA samples from MDS and leukemic phases, obtained four months apart, by both targeted NGS and SNP array revealed swift alterations in the genomic profile, suggesting a linear clonal evolution from MDS to AML (Figure 2). The transition from the mosaic to complete LOH at chromosome 4q13.3q35.2 explains the increase in VAF of the *TET2* pathogenic variant to an almost homozygous status.

Detailed information about the employed methodology is found in the Supplementary Materials.

## 3. Discussion

The detection of RS in BM aspirate of LR-MDS patients has generally been associated with favorable prognosis concerning the overall survival and rate of leukemic transformation, and *SF3B1* mutations are frequently encountered in this condition. However, the patient described did not carry an *SF3B1* mutation and presented BM multilineage dysplasia at diagnosis, the latter being found to represent an independent prognostic marker of aggressive disease [11].

The *TET2* R1440Tfs*38 identified in our patient was previously reported in MDS [12]. Although classified as a tier 2 variant due to the absence of functional studies, it is expected to induce protein truncation by altering the reading frame, and it is predicted to result in a loss of protein function based on the known effects of other downstream truncated mutants, such as S1758X [13].

Acquired abnormalities of the *TET2* gene encoding ten-eleven translocation methylcytosine dioxygenase 2 critically involved in active DNA demethylation are detected in 15–27% of MDS cases. These anomalies may affect one or both *TET2* alleles leading to disabled enzyme function and include frameshift, missense or nonsense pathogenic variants, deletions, and LOH events at the 4q region [14]. *TET2* anomalies were detected in most of the BM cells of MDS patients, including CD34+ progenitors, indicating their early occurrence and involvement in disease initiation, as suspected in our case, too [15]. Additionally, somatic *TET2* variants were found in age-related clonal hematopoiesis, a precursor condition to myeloid neoplasms [16]. As shown in a seminal study that employed *TET2*-knockout (Tet2^−/−)^ mice and mouse exome sequencing, *TET2* loss promoted hypermutagenicity in HSPCs, preferentially in the proximity of CpG sites, leading to hematologic malignancies after long latencies. Thus, *TET2* loss may cause progressive acquisition of additional driver mutations, ultimately resulting in various disease phenotypes [17].

Concerning the prognostic impact of *TET2* abnormalities, in a meta-analysis of 14 studies including 1983 patients, it was shown that the presence of *TET2* mutations did not significantly impact the overall survival in MDS but were associated with higher response rates to HMA [18]. Moreover, MDS-related *TET2* mutations were found to suppress the function of anti-tumor natural killer cells, and HMA could normalize their hypermethylated status and restore their activity [19].

The second genomic aberration detected in our patient at the MDS stage was a serine/arginine-rich splicing factor 2 (*SRSF2*) variant that, according to its VAF, represents a subclonal event. Although this in-frame deletion in the *SRSF2* gene was classified as VUS by ACMG criteria, it was suggested, using both isogenic cell lines and primary patient samples, that *SRSF2* P95_R102del, rarely encountered in MDS, might induce RNA splicing alterations similar to those provoked by the hotspot pathogenic *SRSF2* P95 variants, being likely pathogenic [20]. Somatic mutations affecting RNA splicing machinery are observed in approximately 50% of MDS patients. Among them, *SRSF2* mutations were associated with an increased risk of direct or indirect leukemic transformation [9].

The genetic lesions identified at the moment of MDS conversion into AML were represented by trisomy 13 and likely pathogenic variants of *RUNX1*, *BCOR,* and *BCORL1* genes. Isolated trisomy 13 is a rare event in AML, highly associated with *RUNX1* and *SRSF2* codon 95 mutations and exhibiting an inferior survival. Gene expression analysis indicated an overexpression of *FLT3* and *FOXO1* genes and a downregulation of *SPRY2* tumor suppressor gene, possibly contributing to leukemogenesis [21]. However, as conventional cytogenetics was not performed at the leukemic phase in our patient and trisomy 13 was suggested only by SNP array (gain of 96.67 Mb at 13q11q34), it is possible that this anomaly might not have been isolated, and balanced chromosomal rearrangements, such as translocations and inversions, might have been missed. Mutated *RUNX1* was identified as the main molecular predictive factor for rapid progression in a cohort of LR-MDS patients, leading to an altered antitumor cellular defense [22]. Moreover, a recent in vivo study demonstrated that altered innate immune signaling in cooperation with *RUNX1* mutations induced the leukemic transformation of an MDS-like disease [23]. *BCOR* (BCL6 Corepressor) and *BCORL1* (BCL6 Corepressor like 1) are two homologous genes on chromosome X encoding proteins that are subunits of the Polycomb repressive complex 1.1 involved in the transcriptional repression of active genes through histone modifications [24,25]. *BCOR* and *BCORL1* gene alterations disrupt the repressive function of PRC1, leading to activation of abnormal oncogenic signaling programs [25]. In a study including 354 MDS patients, the frequencies of *BCOR* and *BCORL1* mutations were 4.2% and 0.8%, respectively. It was shown that loss-of-function *BCOR* mutations occurred after epigenetic or splicing factor mutations and represented an independent prognostic factor for poor overall survival in MDS [26]. In adult de novo AML, *BCOR* mutations were reported in 3–5% of cases, their frequency being higher in secondary AML [27]. According to 2022 European LeukemiaNet, AML with *BCOR* mutation is classified in the high-risk category [28].

In our patient, it can be assumed that the leukemic clone emerged from the clone harboring homozygous *TET2* and heterozygous *SRSF2* variants that acquired over a short period of time *RUNX1*, *BCOR*, and *BCORL1* nonsense mutations and trisomy 13. It is likely that TET2 deficiency induced by the homozygous *TET2* variant promoted the leukemic conversion of MDS. As shown previously, a normal TET2 activity decreases the acquisition of secondary mutations in MDS HSPCs and, consequently, precludes MDS progression to AML [29].

According to IPSS-R, the patient was classified in the LR-MDS category at diagnosis (2018) as well as at the follow-up in August 2021. However, the retrospectively calculated IPSS-M (0.72) after obtaining the results of the genetic studies indicated an HR-MDS category [30]. By using this improved scoring system, the patient would have likely benefited from HMA, as previously stated [18]. This case report illustrates the crucial role of molecular-based risk stratification in MDS, especially in real-world settings where, due to economic reasons and limited accessibility to comprehensive NGS-based assays [31], somatic mutations and CNV analysis is restricted to MDS patients that are likely to benefit from allogeneic hematopoietic stem cell transplantation.

Although the direct progression of LR-MDS to secondary AML has been previously characterized [9], our reported case is distinguished by a fast deterioration of hematologic parameters (from pancytopenia with low percentage of BM blasts to hyperleukocytosis with elevated percentage of PB blasts) along with a significant change in the profile of genomic aberrations within four months. We also highlight the acquisition of frameshift/nonsense mutations in *RUNX1*/*BCOR*/*BCORL1* genes and of trisomy 13 on the background of *TET2* loss that represents a state of genomic mutagenicity, as previously shown [17].

We acknowledge that our study has several limitations, such as the lack of DNA samples at the moment of initial diagnosis, unavailability of cytogenetic testing at leukemic conversion that would have likely detected trisomy 13, either as a sole anomaly or in association with other structural chromosomal alterations missed by SNP array, and the absence of non-tumoral tissue control. As recently stated, even in the era of remarkable advancements in NGS-based technologies, conventional karyotyping still represents an indispensable tool in hematological malignancies, complementary to molecular assays, with respect to identifications of balanced translocations, inversions, and balanced insertions [32]. Also, bulk NGS may have missed very small *RUNX1*/*BCOR*/*BCORL1* subclones already present at the MDS stage and, ultimately, the employed NGS panel did not cover other possibly relevant genes for disease transformation. Last but not least, as this study is based on a single patient, the reported observations concerning clonal evolution may not apply to similar LR-MDS patients.

## 4. Conclusions

Analysis of clonal evolution to secondary AML in the reported case of an LR-MDS patient indicates that *TET2* loss, due to a frameshift *TET2* mutation and CN-LOH at 4q, probably drove leukemogenesis through acquisition of *RUNX1*/*BCOR*/*BCORL1* mutations and trisomy 13. The changes in the profile of genomic aberrations occurred within a short interval. As a final remark, we highlight the importance of the combined use of conventional cytogenetics and advanced molecular assays as an essential tool for prognostic evaluation in MDS and also for therapeutic decisions.

## Figures and Tables

**Table 1:** Laboratory parameters during MDS and leukemic phases.

Laboratory Parameters	MDS Diagnosis (March 2018)	MDS Re-Evaluation (August 2021)	AML Post-MDS (December 2021)
Hb (g/dL)	7.3	5.1	7.2
HCT (%)	25.3	19	24.1
WBC (×10^9^/L)	4.2	2.1	102.6
ANC (×10^9^/L)	1.8	0.9	4.1
PLT (×10^9^/L)	62	50	36
PB blasts (%)	0	0	91
BM blasts (%)	2	1–2	Not performed
Ferritin (µg/L)	430	627	850
LDH (U/L)	249	318	920
Serum albumin (g/dL)	4.3	4.5	3.6
Serum creatinine (mg/dL)	1.18	1.15	4.25
Conventional cytogenetics	Normal karyotype	Normal karyotype	Not performed

MDS, myelodysplastic syndrome; AML, acute myeloid leukemia; Hb, hemoglobin; HCT, hematocrit; WBC, white blood cell count; ANC, absolute neutrophil count; PLT, platelets; PB, peripheral blood; BM, bone marrow; LDH, lactate dehydrogenase.

**Table 2:** Genetic variants detected in MDS and secondary AML samples.

Genetic Variants	Present in MDS Sample (VAF)	Present in sAML Sample (VAF)
*TET2* c.4317dup, p.R1440Tfs*38	Yes (53.6%)	Yes (94.1%)
*SRSF2* c.284_307del, p.P95_R102del	Yes (25%)	Yes (50.2%)
*RUNX1* c.504_505del p.G170Efs*42	No	Yes (39%)
*BCOR* c.626C>A, p.Ser209*	No	Yes (85.9%)
*BCORL1* c.4012C>T, p.Arg1338*	No	Yes (86.2%)

MDS, myelodysplastic syndrome; sAML, secondary acute myeloid leukemia; VAF, variant allele frequency.

## Data Availability

The original contributions presented in this study are included in the article. Further inquiries can be directed to the corresponding author.

## Supplementary Materials

The following supporting information can be downloaded at: https://www.mdpi.com/article/10.3390/ijms27104557/s1. References [33,34] are cited in the Supplementary Materials.

## Abbreviations

The following abbreviations are used in this manuscript:

MDS	Myelodysplastic syndrome
LR-MDS	Low-risk myelodysplastic syndrome
HR-MDS	High-risk myelodysplastic syndrome
RS	Ring sideroblast
AML	Acute myeloid leukemia
BM	Bone marrow
PB	Peripheral blood
PBMC	Peripheral blood mononuclear cell
BMMC	Bone marrow mononuclear cell
NGS	Next-generation sequencing
VAF	Variant allele frequency
AMP	Association for Molecular Pathology
ACMG	American College of Medical Genetics and Genomics
SNP	array Single nucleotide polymorphism array
CNVs	Copy number variations
CN-LOH	Copy-neutral loss of heterozygosity
HMA	Hypomethylating agent
HSPCs	Hematopoietic stem and progenitor cells
IPSS-R	Revised International Prognostic Scoring System
IPSS-M	Molecular International Prognostic Scoring System
