# Supplementary material for: Critical Role of Molecular-Based Stratification in Low-Risk Myelodysplastic Syndrome with Direct Progression to Acute Myeloid Leukemia: A Case Report
Source: Int J Mol Sci. 2026 May 19;27(10):4557. doi: 10.3390/ijms27104557 (PMC13207156; doi:10.3390/ijms27104557)
Supplement: Supplementary file 1 [file ijms-27-04557-s001.zip › ijms-4223112-supplementary.pdf]

SUPPLEMENTARY MATERIAL

Material and Methods

Conventional cytogenetics

Two independent cultures were obtained from the bone marrow mononuclear cells at MDS diagnosis (2018), as well as at MDS re-evaluation (August 2021). Metaphase preparation was achieved by standard protocols and G-banded karyotype analysis of at least 20 metaphase cells was performed, with an average band resolution of 450-550 bands per haploid chromosome set. Metaphases were analyzed with Olympus microscope and GenASIs Software (Applied Spectral Imaging, California, USA). The results were interpreted according to the International System for Human Cytogenetic Nomenclature (ISCN 2016 and ISCN 2020, respectively).

Targeted next-generation sequencing (NGS)

Targeted DNA sequencing of MDS patient's samples was performed with TruSight Myeloid Sequencing Panel (Illumina) - an amplicon-based NGS panel that focuses on a genomic region of ~141 kb, being covered the full exonic regions of 15 genes and exonic hot spots of 39 genes strongly associated with myeloid malignancies. According to the manufacturer's guidelines, 50 ng of genomic DNA per sample were used to generate DNA libraries. Targeted sequencing was run on the MiSeq Illumina platform employing the MiSeq Reagent Kit v3. Sequencing data were analyzed using on-instrument software in BaseSpace™ Sequence Hub. FASTQ files were generated and the reads were aligned against the human hg19 reference genome. VariantStudio V2.2 (Illumina) software was used for variant calling and annotation.

The first patient sample generated a total of 915,360 reads passing filter, resulting in an estimated mean coverage of approximately 1,950× across the target region. The second sample yielded 1,738,740 reads passing filter, corresponding to a higher estimated mean coverage of approximately 3,700×.

For the first sample, the depth supports detection of variants with a variant allele frequency (VAF) of approximately 0.15%, while for the second sample, the higher coverage enables detection of variants down to approximately 0.08% VAF.

For interpretation of sequence variants two online tools that automate the Association for Molecular Pathology (AMP) and the American College of Medical Genetics and Genomics (ACMG) guidelines were used, namely Franklin by Genoox (https://franklin.genoox.com/) and GeneBe (https://genebe.net/). The accuracy of variant interpretation provided by Franklin was previously validated by comparing its interpretations with that of the consensus interpretation of the Canadian Open Genetics Repository [1]. Also, GeneBe proved a high correlation with the expert assessments from the ClinGen Evidence Repository in terms of the pathogenicity scores of genetic variants [2].

In the absence of non-tumoral tissue control the known germline polymorphisms were excluded by comparing the identified variants against dbSNP public and gnomAD databases.

Catalog of Somatic Mutation in Cancer (COSMIC) database (http://cancer.sanger.ac.uk) was also interrogated to check the reported somatic variants.

Single nucleotide polymorphism (SNP) microarray analysis

SNP microarray analysis for detection of chromosomal anomalies was performed with the high-resolution genome-wide CytoScan 750K Array run on GeneChip® System 3000 instrumentation platform (Affymetrix, Santa Clara, California). The CytoScan 750K Array contains 750,000 probes including 550,000 unique non-polymorphic probes for detection

of copy number variations (CNVs) larger than 100 kb and 200,000 gene-centric SNPs for identifying copy-neutral loss of heterozygosity (CN-LOH) up to 5Mb across the entire genome. This array ensures the coverage of the exons of 526 genes involved in cancer. A total amount of 250 ng of genomic DNA was digested with NspI restriction endonuclease, ligated to adaptors with T4 DNA Ligase, and amplified with primers that are complementary to the adaptors. PCR products were purified with magnetic beads, fragmented, labeled with biotin, and hybridized to CytoScan 750K microarray chip. Chromosome Analysis Suite (ChAS) v4.2.1 was employed for data analysis. Genomic data were aligned to NCBI Build 37 hg19 reference genome and reanalyzed according to National Center for Biotechnology Information (NCBI), University of California, Santa Cruz (UCSC) Genome Browser, Database of Genomic Variants (DGV), DECIPHER, and Online Mendelian Inheritance in Man (OMIM).

References

1. Mighton, C.; Smith, A.C.; Mayers, J.; Tomaszewski, R.; Taylor, S.; Hume, S.; Agatep, R.; Spriggs, E.; Feilotter, H.E.; Semenuk, L.; et al.; Canadian Open Genetics Repository Working Group. Data sharing to improve concordance in variant interpretation across laboratories: results from the Canadian Open Genetics Repository. *J Med Genet.* **2022**, *59*, 571-578. doi: 10.1136/jmedgenet-2021-107738.
2. Stawiński, P.; Płoski, R. Genebe.net: Implementation and validation of an automatic ACMG variant pathogenicity criteria assignment. *Clin Genet.* **2024**, *106*, 119-126. doi: 10.1111/cge.14516.
